# Setting priorities in primary health care - on whose conditions? A questionnaire study

**DOI:** 10.1186/1471-2296-13-114

**Published:** 2012-11-26

**Authors:** Eva Arvidsson, Malin André, Lars Borgquist, David Andersson, Per Carlsson

**Affiliations:** 1 Department of Medical and Health Sciences, National Centre for Priority Setting in Health Care, Linköping University, Linköping, Sweden; 2 Department of Primary Health Care, County Council of Kalmar, Kalmar, Sweden; 3 Department of Public Health and Caring Sciences - Family Medicine and Preventive Medicine, Uppsala University, Uppsala, Sweden; 4 Department of Medical and Health Sciences, Family Medicine, Linköping University, Linköping, Sweden; 5 Department of Management and Engineering, Division of Economics, Linköping University, Linköping, Sweden

## Abstract

**Background:**

In Sweden three key criteria are used for priority setting: severity of the health condition; patient benefit; and cost-effectiveness. They are derived from the ethical principles established by the Swedish parliament 1997 but have been used only to a limited extent in primary care. The aim of this study was to describe and analyse: 1) GPs', nurses', and patients' prioritising in routine primary care 2) The association between the three key priority setting criteria and the overall priority assigned by the GPs and nurses to individual patients.

**Methods:**

Paired questionnaires were distributed to all patients and the GPs or nurses they had contact with during a 2-week period at four health centres in Sweden. The staff registered the health conditions or health problem, and the planned intervention. Then they estimated the severity of the health condition, the expected patient benefit, and the cost-effectiveness of the planned intervention. Both the staff and the patients reported their overall prioritisation of the patient. In total, 1851 paired questionnaires were collected.

**Results:**

Compared to the medical staff, the patients assigned relatively higher priority to acute/minor conditions than to preventive check-ups for chronic conditions. Severity of the health condition was the priority setting criterion that had the strongest association with the overall priority for the staff as a whole, but for the GPs it was cost-effectiveness.

**Conclusions:**

The challenge for primary care providers is to balance the patients' demands with medical needs and cost-effectiveness. Transparent priority setting in primary care might contribute to a greater consensus between GPs and nurses on how to use the key priority setting criteria.

## Background

Priority setting is necessary in every part of the health care system where needs and demands exceed resources. Priority setting takes place both at an aggregated national or regional level and at an individual clinical level [1-4]. Priority setting in primary health care (PHC) is important because outcomes from PHC have significant implications for health care costs and outcomes in the health system as a whole [5].

**Figure 1:**
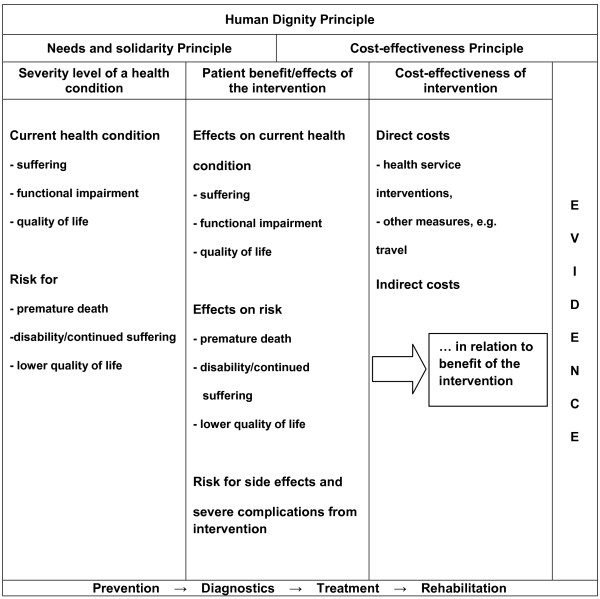
**Schematic description of the key components to be considered in Swedish priority setting**[11] .

Different approaches for priority setting in PHC have been proposed [6-8]. In Sweden, the Government launched a Parliamentary Commission on priority setting in health care, and their final report was published in 1995 [9]. The Swedish parliament ratified the Commission’s proposal in 1997 [10]. One stipulation was that priority setting should be transparent, i.e. the general public should have access to both the results of priority setting decisions and the grounds for them [9,11,12]. All priority setting should be governed by three ethical principles: the human dignity principle, the needs and solidarity principle, and the cost-effectiveness principle. The Government’s bill established that “The relevant issue in prioritisation is that human dignity is not tied to a person’s personal characteristics or functions in society, but to existence itself. It is important to establish that talent, social position, income, age, etc. should not determine who should receive care, or the quality of care” [9]. Hence, the human dignity principle does not tell us how to prioritise, but rather what aspects we are not allowed to consider. In that respect the human dignity principle is applicable in all types of prioritisation situations. To operationalise the principles for practical use, the needs and solidarity principle and the cost-effectiveness principle have been transformed into three key criteria: *severity of the health condition*; *patient benefit*; and *cost-effectiveness of the medical intervention*[11]. The relation between the ethical principles and the criteria, and the variables that should be considered in appraising each criterion, are schematically described in Figure 1. The three criteria are used for priority setting both nationally and regionally in Sweden [13-15]. Several countries with publicly financed health care systems use similar criteria [16,17].

In an earlier study we found that PHC staff viewed the three key priority setting criteria as useful [18]. The study also indicated that the key priority criteria were used differently depending on whether patients had an acute or chronic condition.

However, values in society and in health care are changing worldwide. Patients want to influence their own care, both at an individual and a comprehensive level. They tend to regard health services more as a commodity and have rising expectations and demands on accessibility [19]. In Sweden this coincides with a new funding system for PHC where taxes fund primary health care centres (PHCCs) in proportion to the number of patients linked to the health centre. Hence, at their discretion, patients can affect resource allocation by changing PHCC. (Table 1 lists characteristics of Swedish primary health care). This creates tension between the need for the medical staff to economise, the obligation to follow guidelines and the need to satisfy the patients’ requests. It is challenging for PHC to balance patients’ demands with the expanding need for preventive care of chronic conditions.

**Table 1:** Characteristics of Swedish primary health care

**Financing and ownership**	Most of the primary health care centres are publicly owned and publicly financed through taxes.
**GPs and consultation**	Five years of specialist training is required. About 20% of all specialists are GPs. Three consultations with a specialist per inhabitant and year is average; half of these are with a GP. Consultations with GPs are, on average, 20 minutes.
**Work organisation**	Teamwork dominates. GPs work in close collaboration with district nurses and other health care personnel. Most appointments with the PHCC are preceded by a telephone call to a nurse who decides whether to schedule the patient to see a GP, a nurse, or whether advice by telephone will be sufficient.

Since we found no empirical study addressing priority setting by patients and staff in primary health care, our aim was to study prioritising of individual patients in routine primary care.

### Aims

To describe and analyse:

1) How general practitioners (GPs), nurses, and patients set priorities in routine primary health care (PHC).

2) The association between three key priority setting criteria and the overall priority assigned by the GPs and nurses to individual patients.

## Methods

We conducted the study during a 2-week period in 2004 at four PHCCs in southern Sweden. Paired questionnaires were answered by the patients and GPs or nurses for every patient who contacted (visit or telephone call) the PHCC concerning health problems during the study period.

### Settings and participants

The PHCCs were chosen through purposive sampling. They were located in areas with different populations as regards age and social factors. In total, around 25 000 patients were served by the four PHCCs.

Paired questionnaires were given to all patients (parent or guardian of children) who were in contact with the PHCCs regarding a health problem during the study period, and to the staff they were in contact with. Patients who had telephone contact received and answered the questionnaire by mail. In total 3821 patient contacts were registered. The staff returned 3679 questionnaires (96%), and patients returned 2150 (56%). Written consent was obtained according to the Swedish Act (2003:460) on Ethics Review of Research. From the 2150 patient questionnaires we identified 1851 matched pairs with one questionnaire from staff and one from a patient concerning the same contact. Table 2 lists basic characteristics of the consultations. The 299 non-matched patient questionnaires were largely due to errors in coding of the questionnaires, which made matching impossible. In some cases the reason was that questionnaires from staff were missing.

**Table 2:** Basic characteristics of the consultations, N=1851(%)

		**GP**	**Nurse**
**Type of contact**	**Visits**	32	27
	**Telephone**	7	34
**Patient age**	**65 or less**	28	33
	**Over 65**	11	28
**Patient gender**	**Women**	22	37
	**Men**	17	24

### Questionnaires

The questionnaires were pretested at two of the participating health centres, and minor adjustments were made before the study. First, staff registered the health problem or condition that was the main reason for the visit and the related intervention or measure (e.g. further investigation, medical treatment, or health advice). Second, they used a 3-point rating scale (high, moderate, or low) to estimate the severity of the health condition, the expected patient benefit of the planned intervention, and the cost-effectiveness of the planned intervention.

Finally, using a 10-point scale they assigned an overall priority to the patient by answering the question: *How would you prioritise the patient on a scale of 1 to 10, where 1 is the highest?*

The patients used a similar10-point rating scale to estimate their own overall priority by answering the question: *How important do you think your health care needs are compared to other patients?*

### Groups of health conditions

Two senior GPs (EA and MA) independently sorted out two subgroups from all registered health conditions and interventions. Disagreements were resolved through consensus. The *acute/minor* group consisted of acute conditions and minor and time-limited health problems involving minor signs and symptoms, e.g. mild infections and minor injuries with little or no medical impact from medical interventions. The *chronic stable* group included check-ups for chronic stable conditions that were at risk for future complications, e.g. heart failure, diabetes, COPD, and atrial fibrillation. Health conditions and interventions that we excluded were acute conditions requiring treatment or further diagnostic procedures, e.g. infections such as pneumonia or upper urinary tract infection, exacerbation of chronic conditions, and long-lasting conditions with no or little risk for future complications (Table 3).

**Table 3:** Examples of health problems and related interventions included and not included in the analysis

**Acute/minor (n=343)**	**Chronic/stable (n=223)**	**Not included (n=1285)**
Conjunctivitis	Hypothyreosis without symptoms	Pneumonia or suspected pneumonia
*Advice and possible medical treatment*	*Check up of medical treatment*	*Examination and treatment with antibiotics*
Sore throat, fever below 38.5	COPD, patient smokes	
*Advice by telephone*	*Check up, advice on smoking cessation*	Suspected ischemic heart disease, not acute
Mild abdominal pain		*Examination, and possible further investigation and medical treatment*
*Advice by telephone*	Type 2 diabetes mellitus with complications	
Myalgia or tendinitis, short duration	*Check-up, intensified treatment, possible treatment of complications*	Eczema *Examination and treatment*
*Examination and possible medical treatment or referral to physiotherapist*	Atrial fibrillation, risk factors for thrombosis	Osteoarthritis (hip or knee)
	*Anticoagulant therapy*	*Training instructions, medical treatment*

### Data analysis and statistics

Data from the matched questionnaires (n=1851) were analysed in comparing priority setting by staff and patients. When analysing staff's use of the criteria we used all of the staff's questionnaires (n=3679, Table 4). We used paired Student’s *t*-test to determine the relation between patients’ and staff’s priority setting. Multiple regression analysis was used to study the relationship between the overall prioritisation (dependent variable) and the priority setting criteria (independent variables).

**Table 4:** Multiple regression analyses on prioritisation for all staff, GPs and nurses

	**All staff β (95% CI)**	**GPs β (95% CI)**	**Nurses β (95% CI)**
**Severity of the health condition**	1.18 (1.09-1.28)	1.03 (0.88 - 1.19)	1.25 (1.14 - 1.36)
**Patient benefit**	0.70 (0.59-0.80)	0.68 (0.50 - 0.86)	0.68 (0.54 - 0.82)
**Cost-effectiveness**	0.74 (0.64-0.84)	1.12 (0.94 - 1.30)	0.54 (0.42 - 0.66)
**n**	3679	1489	2190
**R**^**2**^	0.45	0.54	0.40

All independent variables had VIF values below 2.5.

P<0.0001 for all explanatory variables.

To examine if the type of consultation, i.e. acute/minor or chronic stable, affected the impact of each of the three different priority setting criteria on overall priority setting, the regression models included interactions between the predictors and type of consultation. All other two-way interactions were also examined. We made estimates using robust standard errors.

All independent variables were tested for multicollinearity by examining their Variance Inflation Factor (VIF). VIF values ≥ 2.5 were considered to indicate multicollinearity.

For all calculations both the 10-point scale and the 3-point scale were used in "the same direction" (the lower number, the higher estimation of priority and severity/benefit/cost-effectiveness).

The Research Ethics Committee of Linköping University approved this study.

## Results

### Comparison between patients and medical staff

When comparing the patient’s overall priority of the health condition and intervention (or intended intervention), with the GP’s or nurse’s priority of the same clinical situation, we found that patients in general assigned a higher priority than staff did, especially for acute/minor conditions (Table 5). The acute/minor conditions comprised 21% of all contacts, and chronic stable comprised 12%. The greatest difference was found between GPs and patients for acute/minor conditions, where the mean difference was 1.33. The most frequently registered acute/minor condition and intervention was *upper respiratory tract infection* and *medical examination and advice*. The mean overall rating of these patients on the 10-point scale (with 1 being the highest priority and 10 the lowest) was 8.1 by GPs and 5.6 by patients.

**Table 5:** **Overall prioritisation of common health conditions by patients and staff (paired *t*-test, means)**

		** n**	**Staff**	**Patients**	**Difference (95% CI)**	**p**
**All health problems**	**All staff**	1851	5.53	4.75	0.79 (0.65−0.92)	p=<.0001
	**GPs**	718	5.69	4.63	1.05 (0.84−1.26)	p=<.0001
	**Nurses**	1133	5.43	4.82	0.62 (0.44−0.79)	p=<.0001
**Acute/minor health conditions**	**GPs**	169	6.02	4.69	1.33 (0.91−1.76)	p=<.0001
	**Nurses**	174	6.02	4.83	1.19 (0.74−1.64)	p=<.0001
**Chronic stable health conditions**	**GPs**	84	4.76	4.82	−0.06 (−0.63−0.51)	p=0.835
	**Nurses**	139	5.67	5.01	0.65 (0.19−1.12)	p=0.006

One of the most frequently registered chronic conditions and interventions was *yearly check-up for ischemic heart disease* where the mean ratings were 4.1 by GPs and 4.6 by patients.

### Use of priority setting criteria

The estimations of the three key criteria were associated with the overall prioritisation of each patient; the coefficient of determination 0.54 was for GPs and 0,40 for nurses (Table 4). In the multiple regression analysis, when analysing GPs and nurses together, severity of the health condition was the priority setting criterion that had the strongest association with their overall prioritisation of the patients, followed by cost-effectiveness and patient benefit (Table 4). When analysing GPs and nurses separately, we found that the criterion that had the strongest association with the overall prioritisation for the GPs was cost-effectiveness. For the nurses it was severity of the health condition.

An interaction analysis showed an interaction between the severity of the condition and the cost-effectiveness of the intervention for GPs. If both were scored low, then the overall priority was not as low as it would have been without the interaction effect.

Interactions between type of condition and the independent variables were tested to determine if the three key criteria were weighted differently depending on whether the condition was acute/minor or chronic stable. Only one interaction was found. For nurses, patient benefit was more important if the patient had a chronic stable condition rather than an acute/minor one.

## Discussion

The central finding was that patients, compared to medical staff, gave higher priority to acute/minor conditions than to chronic conditions and preventive measures when they prioritised individual patients in routine primary care. Of the three criteria used by the staff in priority setting, the severity of the health condition had the strongest association with overall priority. For GPs alone cost-effectiveness had the strongest association.

### Comparison between patients and medical staff

This initial study of prioritisation of individual patients in routine care in general practice indicates that GPs, nurses, and patients hold different opinions on what type of health conditions and interventions should receive highest priority. GPs generally gave higher priority to patients with chronic stable conditions where the focus was on trying to prevent future complications, while patients gave the highest priority to acute/minor health problems. An earlier study showed that patients in PHC have high expectations on the health service to meet all of their demands, including health care for trivial problems [20]. Different opinions between GPs and patients on what is most important have also been found in other studies [21,22]. This disagreement between needs as defined by patients and by physicians might be explained by their different viewpoints; for GPs medical knowledge is an important factor in the priority setting process. Even if the GPs also consider factors other than biomedical criteria they emphasise the *medical perspective* in priority setting [18,23,24]. In our earlier study the GPs acknowledged the medical, evidence-based, perspective concerning the effect of secondary prevention in chronic stable conditions compared to interventions in self-limiting disorders [18]. This might explain the high priority given to check-ups of patients with chronic conditions. It seems to be reasonable that patients are more influenced by their present symptoms than by the future risk of complications.

### Use of priority setting criteria

The association between the three key criteria and the overall priority indicates that the criteria largely influenced the overall prioritisation of each patient, for both the GPs and the nurses, which confirms the results from our earlier study where the GPs and nurses reported that the criteria were useful in day-to-day priority setting [18].

Use of the three criteria, especially cost-effectiveness, differed between doctors and nurses in their overall prioritisation. Other studies show that nurses and GPs found cost-effectiveness difficult to understand and apply [25,26]. Formal health economic evaluations are seldom available for health conditions and interventions common in primary care. In one study, GPs described how they tried to make a rough estimate of cost-effectiveness to use as a basis for priority setting [27]. Our previous study found that GPs and nurses made an assessment of anticipated benefits or cost-effectiveness for the individual patient by thinking of a group of similar patients [18].

Nevertheless, cost-effectiveness was the criterion having the greatest influence on overall priority for the GPs. This contrasts with the original proposal from the Priorities Commission, which ranked the cost-effectiveness principle as the lowest of the three ethical principles. According to the Commission, the cost-effectiveness principle should be applied only when comparing methods of treatment for the same disease, since the effects cannot otherwise be compared in an equitable way. However, the Government states in its bill “…it is essential to differentiate between the cost-effectiveness of a treatment for a particular individual and that for health care at large. A cost-effectiveness principle that concerns choices between different interventions for the individual patient must be applied as proposed by the inquiry, and is subordinated to the principles of human dignity and needs and solitary. Nevertheless, it is essential for health services to strive for high cost-effectiveness as regards health care services in general” [9]. Here the Government indicates a different rank of cost-effectiveness in priority setting between the individual and group levels. In practical use, e.g. by the Swedish National Board of Health and Welfare and the Dental and Pharmaceutical Benefits Agency, cost-effectiveness plays a central role in writing national guidelines for priority setting and in decisions regarding which pharmaceuticals the state will subsidise. Still, we have little information about how the priority setting principles are actually applied at the individual level. It is possible that the new Swedish funding system have increased cost awareness among GPs since PHCCs have local responsibility for a limited budget that must cover everything, including drugs, for their patients [28]. GPs are also becoming more familiar with economic evaluations through the national guidelines on priority setting [29].

Patient benefit had the least influence on the GPs’ overall priority. This contrasts to another study concerning prioritisation of new technology by committees where the general public, patients, health professionals, and administrators participated. In this study, patient benefit was the most important factor for decisions [30]. Patient benefit is a subset of cost-effectiveness. However, in this study multicollinearity of the predictors in the regression analysis was negative, implying no association. For nurses, cost-effectiveness was the least important criteria. In a focus group, nurses in the study said they did not want to think about the costs of health care at all [18]. However, the nurses evaluated patient benefit as more important for patients with chronic stable conditions than for patients with acute/minor conditions.

Severity is a familiar concept in routine PHC work and is used as an established criterion for priority setting also in other countries [16,17]. For the GPs in our study, estimated severity had a slightly smaller effect on overall priority than cost-effectiveness, and for the nurses severity influenced overall priority much more than the other two criteria.

### Strengths and limitations

The response rate from staff was high (96%). However, the lower response rate from the patients (56%) was considered to be acceptable. Similar rates have been reported in comparable types of studies, and moreover response rates in questionnaire studies are generally declining [21,31]. Responders and non-responders did not differ concerning age and gender, but telephone contacts were higher among the non-responders. We do not know if this affected the results.

The large number of observations is a strength of this study. However, despite over 1800 complete pairs of observations, the frequency of each specific health condition and intervention was low due to the wide variation of health problems in primary care [32].

A weakness is the lack of an established classification system for health problems and related interventions. The two groups, a) acute/minor time limited conditions and interventions and b) chronic stable conditions with a risk for future complications, may be defined differently. What the groups include or exclude is not clearly specified. To make the groups as well-defined as possible, we included only typical conditions.

Comparison of estimated values on an ordinal scale can cause problems. First, the scales are subjective and different persons may interpret them differently, which can make comparisons hazardous. Second, there is a tendency to avoid using the ends of the scales in subjective judgements where responders have some doubts about “proper” answers [33]. In our study this especially applied to patients who often responded around the mid-point of the priority scale. This *central tendency bias* might have affected the result so that differences in prioritising, measured in scale-points, can be relatively small. Hence, the direction of the differences, or the relation between ratings, might be more interesting than the actual numbers.

The staff were supposed to fill in the questionnaire directly after each consultation. We selected a simple three-step ordinal scale to make the study feasible in day-to-day care. Both the 10- and the 3-point scales used in this study are used in Sweden on the national and regional levels for priority setting. In recent years, 4-point scales have been used. Since it is difficult to find objective mathematical or quantitative methods to calculate priority levels, qualitative estimations are usually used [11,34,35].

The rating on the 3-point scales was introduced in the regression model as an interval scale since the variables had a linear approximation with our dependent variable. Introducing the variables with dummy coding made negligible differences in the results.

The lack of association between patient benefit and cost-effectiveness found in this study, suggests that patient benefit and cost-effectiveness were seen as distinct from each other by the staff. It is possible that the staff did not fully understand the concept of cost-effectiveness, but mixed it up with costs per se.

As organisational characteristics and professional roles in PHC differ between countries, some of the findings might be context-bound to Sweden. Since this might be a limitation of the study, further studies are needed in other settings.

### Health policy implications

The results of this study of individual patients may have implications for development of priority setting in PHC at the national or regional level. The high influence that GPs gave cost-effectiveness in their priority setting might influence prioritising and rationing for individual patients in day-to-day primary care in a different way than the policy makers originally intended.

Comprehensiveness, continuity, and person-centredness are essential to better health outcomes in PHC. Close and trusting relationships with GPs and nurses who know their patients are critical for a well-functioning PHC [19]. There is ample evidence that continuity of care in PHC contributes both to better quality of care and better outcomes [36,37]. Early detection and prevention of problems are facilitated. Furthermore, episodes of care that begin with visits to an individual’s primary care clinician, as opposed to other sources of care, are associated with significantly lower costs [38].

Also, single consultations for minor problems might yield high patient benefit and cost-effectiveness in the long term and might therefore be acknowledged by the GPs and nurses. On the other hand, there is a risk that the adaptation of priority setting to the patients’ demands, rather than needs, might influence consumption and funding of health care in an unfair and inefficient way [19]. Without a well-functioning system for priority setting there is a risk that preventive care for chronic conditions with few overt symptoms gets forced out in favour of minor self-limiting problems. In recent years, the Swedish government has focused on accessibility in health care. National figures are presented regularly on the number of days patients must wait for an appointment in primary care. Trends indicate that the number of visits are increasing and waiting times are decreasing. This, in combination with the new Swedish funding system for primary care – where patients direct and redirect funds by their choice of PHCC – might make prioritising according to ethical principles difficult. For instance, a study indicated that waiting-time guarantees led health care providers to give priority to access rather than needs for care [39].

The challenge for primary care providers is to balance the patients’ demands with their medical needs. Patients in PHC have some acceptance of rationing [20,40,41], and the legitimacy of policy decisions depends less on total consensus than on procedural fairness and transparency [42]. Systematic, transparent, priority setting in PHC – where decisions and the grounds for them are accessible to everyone – might also increase the consensus between GPs and nurses on how to use the key priority setting criteria and prevent other values from overshadowing them [43].

## Conclusions

Patients, compared to medical staff, gave relatively higher priority to acute/minor conditions than to preventive check-ups for chronic conditions when they prioritised individual patients in routine primary care. Of the three priority setting criteria, *cost-effectiveness* had the greatest impact on overall priority for GPs while *severity* had the greatest impact for nurses.

The challenge for primary care providers is to balance the patients’ demands with their medical needs. Systematic, transparent, priority setting in PHC might contribute to a greater consensus between GPs and nurses on how to use the key priority setting criteria. Studies on the extent to which such work is done might promote greater understanding for priority setting in PHC.

## Competing interests

The authors declare that they have no conflict of interests.

## Authors’ contributions

EA, MA, LB and PC planned the study. EA conducted the first analysis in dialogue with all in the research group. DA made the statistical analysis and calculations. All authors performed the final analysis, and were involved in drafting the manuscript as well as the final approval of the manuscript.

## Pre-publication history

The pre-publication history for this paper can be accessed here:

http://www.biomedcentral.com/1471-2296/13/114/prepub
